# Is intensive counseling in maternity care feasible and effective in promoting physical activity among women at risk for gestational diabetes? Secondary analysis of a cluster randomized NELLI study in Finland

**DOI:** 10.1186/1479-5868-9-104

**Published:** 2012-09-05

**Authors:** Minna Aittasalo, Jani Raitanen, Tarja I Kinnunen, Katriina Ojala, Päivi Kolu, Riitta Luoto

**Affiliations:** 1 The UKK Institute for Health Promotion Research, P.O. Box 30 33501, Tampere, Finland; 2 Tampere School of Public Health, University of Tampere, Tampere, Finland; 3 National Institute for Health and Welfare, Helsinki, Finland

**Keywords:** Physical activity, Counseling, Pregnancy, Intervention, Effectiveness, Maternity care, Gestational diabetes

## Abstract

**Background:**

Women who are physically active during early pregnancy have notably lower odds of developing gestational diabetes than do inactive women. The purpose of the intervention was to examine whether intensified physical activity (PA) counseling in Finnish maternity care is feasible and effective in promoting leisure-time PA (LTPA) among pregnant women at risk of gestational diabetes.

**Methods:**

Fourteen municipalities were randomized to intervention (INT) and usual care group (UC). Nurses in INT integrated five PA counseling sessions into routine maternity visits and offered monthly group meetings on PA instructed by physiotherapists. In UC conventional practices were continued. Feasibility evaluation included safety (incidence of PA-related adverse events; questionnaire), realization (timing and duration of sessions, number of sessions missed, attendance at group meetings; systematic record-keeping of the nurses and physiotherapists) and applicability (nurses’ views; telephone interview). Effectiveness outcomes were weekly frequency and duration of total and intensity-specific LTPA and meeting PA recommendation for health self-reported at 8-12 (baseline), 26-28 and 36-37 weeks’ gestation. Multilevel analysis with adjustments was used in testing for between-group differences in PA changes.

**Results:**

The decrease in the weekly days of total and moderate-to-vigorous-intensity LTPA was smaller in INT (N = 219) than in UC (N = 180) from baseline to the first follow-up (0.1 vs. -1.2, p = 0.040 and −0.2 vs. -1.3, p = 0.016). A similar trend was seen in meeting the PA recommendation (−11%-points vs. -28%-points, p = 0.06). INT did not experience more adverse events classified as warning signs to terminate exercise than UC, counseling was implemented as planned and viewed positively by the nurses.

**Conclusions:**

Intensified counseling had no effects on the duration of total or intensity-specific weekly LTPA. However, it was able to reduce the decrease in the weekly frequency of total and moderate-to-vigorous-intensity LTPA from baseline to the end of second trimester and was feasibly embedded into routine practices.

**Trial registration:**

ISRCTN 33885819 ( http://www.isrctn.org)

## Introduction

The health benefits of physical activity (PA) among the general population are well documented. For cardiovascular health an accumulation of a minimum of 150 min of at least moderate-intensity PA spread evenly over at least three days of the week is recommended [1]. In Finland only half of the adult population meets this recommendation [2].

The general PA recommendation for health is also valid during uncomplicated pregnancy [1,3,4]. However, during pregnancy women’s PA tends to decrease [5,6] and shift from moderate to light-intensity [5,7,8] due to, for example, physical limitations [9,10] and fear of harming the fetus [10,11].

Moderate-intensity PA has not been shown to be deleterious to pregnancy outcome [12-14] but, instead, is associated with a number of benefits for both mother and fetus [15-17]. Examples of maternal advantages are improved cardiovascular function, limited pregnancy weight gain, decreased musculoskeletal discomfort, reduced incidence of muscle cramps and lower limb edema, improved mood stability and attenuation of gestational hypertension and gestational diabetes [15].

Today, special interest focuses on gestational diabetes, which is the most common complication of pregnancy [18] and may later in life lead to a 71% higher incidence rate of type 2 diabetes in mothers with gestational diabetes than in mothers with no gestational diabetes [19]. Gestational diabetes can also cause fetal macrosomia, birth trauma to mother and baby, induction of labor or caesarean section and transient neonatal morbidity, neonatal hypoglycemia, perinatal death and obesity and/or diabetes developing later in the baby’s life [20]. A recent meta-analysis suggests that women who are physically active during early pregnancy may have 24% lower odds of developing gestational diabetes than inactive women [21].

Promoting PA during pregnancy among women at risk for gestational diabetes is therefore important. Concern about the health of the babies may also make women more receptive to health education and behavioral modifications during pregnancy than when not pregnant [18,22,23]. Studies show that receiving advice from health professionals in early pregnancy may be an important predictor of regular exercise in late gestation [24,25] and pregnant women may need information and encouragement especially in engaging to moderate-to-vigorous-intensity PA [26,27]. Finnish maternity care provides a favorable frame for behavior modification enabling individual counseling and frequent support with an average number of 16 personal contacts with a nurse or physician during pregnancy [28]. It is also free of charge and utilized by practically all pregnant women [29].

To date, no studies have been published on the effects of counseling on the PA behavior of pregnant women at risk for gestational diabetes. The studies aiming to prevent gestational diabetes have mostly used structured exercise e.g. [30-34]. One exception is the ongoing study by Chasan-Taber et al. [35], which is based on one face-to-face counseling session in hospital, use of pedometers and activity logs and follow-ups by mail and telephone.

The purpose of the present study was to examine whether intensified PA counseling in Finnish maternity care is feasible and effective in promoting leisure-time PA (LTPA) among pregnant women at risk for gestational diabetes. The study is part of a larger intervention aiming to prevent gestational diabetes through lifestyle counseling [36]. The effects of the intervention on gestational diabetes and on other lifestyle issues have been reported elsewhere [37].

## Methods

### Participants

The complete description of study design and methods can be found in a separate article [36]. The study was approved by the Ethical Committee of Pirkanmaa Hospital District. Fourteen municipalities from South West Finland and 53 public health nurses working in the maternity clinics of the municipalities participated in the study. The municipalities were arranged into pairs, which were matched for the size and socioeconomic level of the population, annual number of deliveries, incidence of gestational diabetes and neighborhood pattern (urban/rural) obtained from public registries and estimations provided by the municipalities.

The municipalities were randomized in pairs to intervention (INT, N = 7) and usual care group (UC, N = 7). As the pregnant women contacted their maternity clinic, which was determined by their place of residence, to set the first appointment at 8-12 weeks’ gestation, the public health nurses in INT (N = 23) and in UC (N = 30) recruited those with at least one risk factor for gestational diabetes (BMI ≥ 25 kg/m2, gestational diabetes or any signs of glucose intolerance or macrosomic newborn (≥ 4500 g) in any earlier pregnancy, type 1 or 2 diabetes in first or second grade relatives, age ≥ 40 years). The recruitment period lasted from October 2007 to December 2008. Women were excluded if they were under 18 years of age, unable to speak Finnish, had multiple pregnancy, gestational or type 1 or 2 diabetes, physical restriction preventing PA, substance abuse, treatment or clinical history of any psychiatric or other illness.

### Physical activity counseling

#### Intervention group (INT)

The structure and topic of PA counseling were based on the model by Laitakari and Asikainen [38], which combines a logical sequence of work steps for health care purposes, personal aspects, stage of adoption, determinants of PA, educational concepts and strategies for maintenance. The procedure has been found feasible in Finnish maternity care among first time pregnant women not screened for any specific health risks [39].

One primary and four booster PA counseling sessions were integrated to five of the routine visits to a public health nurse. The most important rationale for the timing and the number of sessions was the transferability of the intervention to routine practices after the study. Therefore the sessions were integrated only into normal check-up visits, not into the visits involving special procedures such as ultrasound or a physician contact. The nurses were trained for the PA counseling procedure at one full-day session arranged by the researchers. The primary counseling session was to take place at 8-12 weeks’ gestation and the subsequent booster sessions at 16-18, 22-24, 32-34 and 36-37 weeks’ gestation. The allocated time for the primary session was 20-30 min and for each of the booster sessions 10-15 min. The structure and the topics of the sessions were guided by a counseling manual, which the nurse completed for each participant at each session. The counseling manual followed the principles of the counseling model (38). At the primary session, the participant’s current LTPA, its sufficiency for health as well as the benefits, barriers, incentives, readiness and goals of LTPA as well as indications to stop PA were discussed with the help of a take-home leaflet designed for pregnant women. The nurse then assisted the participant to make a weekly action plan including LTPA modes and their frequency, duration and RPE (ratings of perceived exertion)-based intensity between 6-20 [40]. The focus was on LTPA of RPE ratings 12-14 (somewhat hard) as suggested in the PA guidelines for pregnant women [3,4,41].

The minimum weekly LTPA dose entered progressively into the action plan was 800 MET (multiples of resting metabolic equivalents) minutes. This was determined as a result of the following procedure: During the planning phase of the study the CDC-ACSM publication [42] was the most recent PA recommendation for cardiovascular health and it had also been deemed valid during uncomplicated pregnancy [3,4,41]. Based on the recommendation it was estimated that a minimum of 40 min of moderate-to-vigorous-intensity PA [7 METs] 3 times per week equaling 840 weekly MET minutes was needed for fitness and 30 min of moderate-intensity PA [5 METs] on 5 weekdays equaling 750 weekly MET minutes was needed for health. Both aspects, health and fitness, were considered because some women may have preferred to continue their vigorous LTPA during pregnancy. As a result, a consensus of a weekly minimum of 800 MET minutes was drawn from the estimates. After the initiation of the study the PA recommendation for the general adult population was updated suggesting 750 MET minutes of *moderate-intensity* PA as the upper limit of minimum weekly PA dose for cardiovascular health [43]. This indicates that the consensus of 800 weekly MET minutes also *including light-intensity* LTPA due to participants’ different LTPA backgrounds was quite appropriate.

After completing the action plan with the participant the nurse ensured that it included the weekly minimum of 800 MET minutes. In calculations RPE 6-11 equaled three METs, 12-14 five METs and 14-20 seven METs [4,44]. After the visit the participant kept a record of her compliance by making entries in her logbook. Each booster session started with a discussion about how the activity plan had been realized by using the logbook. If the logbook showed that some parts of the action plan had not been realized, the plan was revised for the next visit. Then, also, the weekly MET minutes were recalculated accordingly. The five action plans with logbooks comprised the participant’s follow-up notebook, which was used only for counseling and not for outcome purposes.

At the primary counseling session the participants in INT were offered an opportunity to attend monthly thematic meetings on PA. The meetings were designed especially for the study and arranged after working hours in nearby maternity clinics. The purpose of the meetings was to reinforce PA counseling by providing information and social support for behavior change and by introducing various ways of being physically active. Meetings on five different themes were arranged on a non-stop basis. The dates of all the meetings were provided to the participants at the primary counseling session. Participants attended the first theme following their primary counseling session but eventually participated in all the themes during the course of their pregnancy. The duration of each meeting was two hours: 30 min for getting acquainted, 30 min for the theoretical basis related to the theme and 1 h for the group exercise related to the theme. In all the meetings RPE was used for assessing the intensity of exercise.

The themes of the meetings were: 1) PA during pregnancy; benefits, recommendations, home exercise training (a take-home poster on home exercises during pregnancy), 2) walking; technique, footwear, training outdoors, 3) walking; pedometer, Nordic walking, training outdoors, 4) urinary incontinence; physiology, prevalence, treatment, functional training, 5) postpartum PA; benefits, recommendations, integrating PA into family life, home exercise training (a take-home poster on postpartum home exercises).

The physiotherapists of local health care centers or private clinics conducted the meetings. A week prior to each meeting a SMS reminder was transmitted to all participants. A week after the meeting the instructor contacted all the participants by telephone to encourage them to continue with their weekly action plans and to get feedback on the meeting from those who had attended. The instructors were trained and provided with all the material needed for the theoretical and practical parts of the thematic meetings. Also, they were paid for the time needed for the training, for the actual meetings and for making the telephone calls. The instructors used structured forms to keep record on participants’ attendance at the meetings and of the telephone calls.

#### Usual care group (UC)

Former PA counseling practices were continued in UC. According to our pilot study most nurses discussed PA at the first maternal visit [39]. However, the mean duration of discussions was short, only 7.5 min ranging from 4 to 13 min. The topics raised most frequently were pregnancy related physiological changes and existing PA habits.

### Evaluation

#### Feasibility

Three components were included in the feasibility evaluation: safety, realization of counseling and nurses’ views on applicability. The indicators and evaluation methods regarding the components are described in Table 1.

**Table 1:** Feasibility evaluation of the physical activity (PA) counseling procedure

**Component and indicator**	**Evaluation method**
**Safety**	
Self-reported occurrence of adverse events^1)^ during and immediately after PA in INT^2)^ and UC^3)^	Elicited by the nurses from all the participants during booster visits at 16–18, 22–24, 32–34 and 36–37 weeks’ gestation:
	Have you had any of the following symptoms after the previous visit?
	List of warning symptoms^1)^ and response alternatives per each symptom: 1 = No, 2 = Sometimes, 3 = Often.
	Have you had these symptoms during or immediately after physical activity?
	1 = No, 2 = Yes, which symptoms? ___________________
	The first question has been reported previously [37]. The latter question was used in this study to indicate adverse events during and immediately after PA.
**Realization of counseling in INT**^2)^	
Timing of the PA counseling sessions	A specific space was provided in the counseling manual for the nurses to enter weeks’ gestation regarding each counseling session in INT.
Duration of the PA counseling sessions	A specific space was provided in the counseling manual for the nurses to enter the time when each counseling session started and ended.
Number of PA counseling sessions missed	Nurses’ notes in the counseling manual under the session in question indicated that the session was completed. No notes indicated a missed session.
Attendance to physical activity thematic meetings^4)^	Participation lists of the instructors.
**Applicability of counseling in INT**^2)^	
Applicability of the PA counseling sessions to routine maternity visits viewed by the nurses.	A 5-point scale in a structured form used in interviewing the nurses by telephone after the study (1 = inapplicable … 5 = very applicable).

^1)^ Nausea, vaginal bleeding, painful contractions, dizziness, dyspnea, headache, chest pain, profound fatigue or weakness and calf pain or swelling classified as warning signs to terminate exercise during pregnancy [3,4].

^2)^ Intensified PA counseling, intervention group.

^3)^ Conventional PA counseling, usual care group.

^4)^ Participation in all and in at least 3 of the total 5 meetings.

#### Effectiveness

A baseline LTPA questionnaire at 8-12 weeks’ gestation and two follow-up questionnaires at 26-28 and 36-37 weeks’ gestation were self-administered by the participants in INT and UC to compare the group differences in changes in the weekly number of days and minutes of total and intensity-specific (moderate-to-vigorous, light) LTPA as well as in meeting the PA recommendations for health by spreading a minimum of 150 min of moderate-to-vigorous-intensity LTPA over at least 3 days a week [1]. The validity and repeatability of the questionnaire have been reported earlier [45].

The questionnaire was modified from the International Physical Activity Questionnaire (IPAQ) ( http://www.ipaq.ki.se) but several adaptations were made to better distinguish the structured and unstructured features of PA. The questionnaire included two basic PA domains: 1) LTPA excluding household chores indoors and outdoors and 2) household chores indoors and outdoors only. Occupational PA was excluded because the intervention targeted only leisure physical activity.

Weekly number of days and average minutes per day of domain-specific LTPA at three intensity levels were elicited. The intensities were described as degree of breathlessness (none, some, marked) because the expressions “light”, “moderate” and “vigorous” may be difficult to understand for some people [46]. In the analysis the categories “moderate” and “vigorous” were combined. The weekly number of minutes spent in intensity-specific LTPA was calculated by multiplying the minutes per day by the weekly number of days.

### Statistical methods

Descriptive information is given as arithmetic means, standard deviations (SD) and percentages. Group differences were based on STATA software (version 11.0) multilevel analysis, which made it possible to examine the simultaneous influences of four different levels - pair, municipality, nurse and individual - on outcomes and helped to correct the results for intra-level variation. Xtmixed (multilevel mixed-effects linear regression) command was used for continuous variables (weekly number of days and minutes of total and intensity-specific LTPA) and GLLAMM (generalized linear latent and mixed models) command for binary outcome (meeting the PA recommendation for health, self-reported occurrence of adverse events during or immediately after PA). Four-level random effects were fitted to the commands.

Individual-level adjustments included in the models were the baseline value of each specific PA outcome, age (continuous), pre-pregnancy body mass index (BMI) based on self-reported weight and height at 8-12 weeks’ gestation (<25, ≥25), smoking status before or during pregnancy (no/yes), primiparity (no/yes), education (basic, polytechnic, academic) and working fulltime (no/yes). The effects of the intervention on continuous variables are indicated as coefficients (Coeff.) and for binary variable as odds ratios (ORs). For both, 95% confidence intervals (CI) and statistical significance levels (p-values) are reported.

## Results

### Subjects

**Figure 1:**
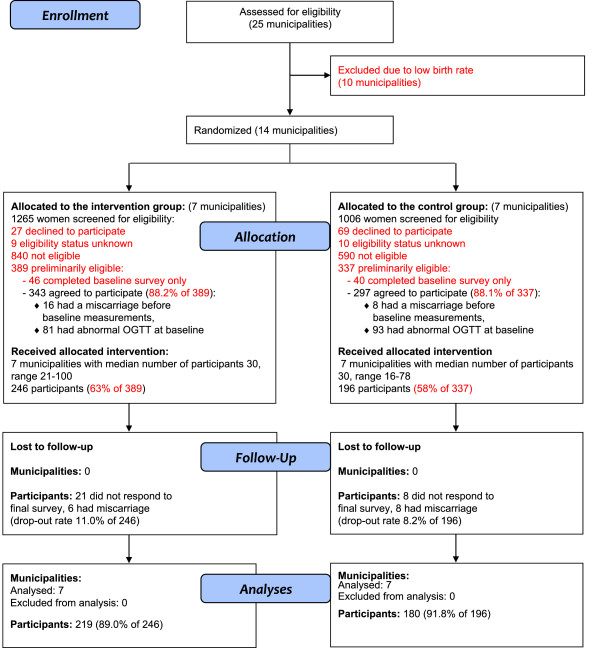
Participant flow of the study.

Of the 2,271 women contacting maternity care during the recruitment period altogether 726 (32%) were preliminary eligible for the study (Figure 1). Of these, 86 (12%) agreed only to complete the baseline questionnaire and 640 (88%) volunteered for the intervention. However, 24 of the volunteers miscarried before the initiation of the study and 174 already had pathological oral glucose tolerance test at baseline and were thus excluded as ineligible for the study. From the 442 participants (246 in INT and 196 in UC) receiving the intervention 14 miscarried (6 in INT, 8 in UC) after initiation of the study and 29 did not respond to the final survey. This left 399 participants (90% of 442) in the final sample, of whom 219 (89% of 246) were in INT and 180 (92% of 196) in UC.

There were fewer women among the participants than among all eligible women with baseline information (N = 726), whose BMI exceeded 25 (61% vs. 66%) and who smoked before and/or during pregnancy (26% vs. 30%). There were more women among participants compared to dropouts with polytechnic or academic education (66% vs. 27%). Also, the participants were more likely than the dropouts to be primiparous (44% vs. 28%), smokers before or during pregnancy (23% vs. 0%), working fulltime (63% vs. 55%) and having more days per week with moderate-to-vigorous-intensity PA (3.9 vs. 3.4). Baseline characteristics of the participants in INT and UC are presented in Table 2. As can be seen from Table 2, some baseline differences also existed between INT and UC: the participants in INT were more often than their counterparts in UC academics, primiparous, full-time workers, non-smokers and less physically active. Consequently, these differences were taken into account in analyzing the between-group differences in PA changes.

**Table 2:** Baseline characteristics of the participants in the intervention (INT) and usual care (UC) group

	**INT**	**UC**
	**(N = 219)**	**(N = 180)**
Age in years, mean (SD)	29.5 (4.77)	30.0 (4.68)
Primiparous - N (%)	103 (47.0)	73 (40.6)
Body mass index (BMI, kg/m^2^) before pregnancy, mean (SD)^1)^	26.3 (4.9)	26.4 (4.3)
Range of BMI before pregnancy^2)^	17.0 - 48.5	17.2 - 37.8
Education		
Academic, N (%)	58 (26.8)	36 (20.6)
Polytechnic, N (%)	85 (39.4)	80 (45.7)
Basic or secondary school, N (%)	73 (33.8)	59 (33.7)
Working fulltime, N (%)	147 (67.1)	104 (57.8)
Smoking before and/during pregnancy, N (%)^3)^	44 (20.9)	45 (26.2)

^1)^ Based on pre-pregnant weight and height self-reported in the baseline questionnaire.

^2)^ Outliers of 48.5 and 40.4 excluded from the statistical analysis.

^3)^ Information missing N = 16.

### Feasibility

The data on adverse events during or immediately after PA at 16-18, 22-24, 32-34 and 36-37 weeks’ gestation was available from 90.9%, 88.6%, 85.8 and 78.5% of the participants in INT and from 98.3%, 63.9%, 62.2% and 60.0% of the participants in UC respectively. Approximately one third of the participants in INT and UC had experienced at least one adverse event during or immediately after PA during the course of their pregnancy (Table 3). No between-group differences were detected in the occurrence of at least one adverse event adjusted for possible confounders. In terms of symptom-specific events, those most frequently reported by the participants at 16-18, 22-24, 32-34 and 36-37 weeks’ gestation were dyspnea (10.3%, 10.2%, 14.6%, 18.9%), musculoskeletal symptoms (10.8%, 14.0%, 19.6%, 20.5%) and miscellaneous (13.8%, 22.7%, 24.8%, 22.4%). Throughout the pregnancy dyspnea was reported more often in UC than in INT whereas musculoskeletal symptoms and painless contractions were more common in INT than in UC. Painful contractions occurred more often in UC than in INT especially from 32-34 weeks’ gestation onwards. The number of miscarriages after the initiation of the intervention was 6 (1.8%) in INT and 8 (2.7%) in UC.

**Table 3:** **Self-reported occurrence of adverse events**^**1)**^**during and immediately after physical activity in the intervention (INT) and the usual care group (UC) at the follow-up visits **

**Adverse event**	**16-18 weeks’ gestation N (%)**		**22-24 weeks’ gestation N (%)**		**32-34 weeks’ gestation N (%)**		**36-37 weeks’ gestation N (%)**	
	**INT**	**UC**	**INT**	**UC**	**INT**	**UC**	**INT**	**UC**
	**N = 199**	**N = 117**	**N = 194**	**N = 115**	**N = 188**	**N = 112**	**N = 172**	**N = 108**
Nausea	7 (3.5)	-	2 (1.0)	-	1 (0.5)	-	1 (0.6)	-
Vaginal bleeding	-	-	-	-	1 (0.5)	-	-	1 (0.9)
Painful contractions	-	-	-	4 (3.5)	-	8 (7.1)	1 (0.6)	7 (6.6)
Dizziness	6 (3.0)	3 (2.6)	4 (2.1)	1 (0.9)	1 (0.5)	3 (2.7)	1 (0.6)	-
Dyspnea	7 (3.5)	8 (6.8)	3 (1.5)	10 (8.7)	4 (2.1)	14 (12.5)	7 (4.1)	16 (14.8)
Headache	1 (0.5)	-	1 (0.5)	-	-	-	2 (1.2)	-
Chest pain	-	-	-	-	-	-	-	-
Profound fatigue or weakness	-	1 (0.9)	-	4 (3.5)	2 (1.1)	2 (1.8)	-	2 (1.9)
Calf pain or swelling	-	-	2 (1.0)	1 (0.9)	1 (0.5)	1 (0.9)	-	1 (0.9)
Musculoskeletal symptoms	13 (6.5)	5 (4.3)	17 (8.8)	6 (5.2)	25 (13.3)	7 (6.3)	24 (14.0)	7 (6.5)
Painless contractions	4 (2.0)	-	10 (5.2)	-	29 (15.4)	2 (1.8)	18 (10.5)	2 (1.9)
Cramps	1 (0.5)	-	1 (0.5)	-	2 (1.1)	-	-	-
Miscellaneous^2)^	12 (6.0)	8 (7.8)	22 (11.3)	13 (11.4)	23 (12.2)	14 (12.6)	16 (9.3)	14 (13.1)
At least one adverse event	59 (30.1)	23 (20.2)	61 (31.9)	32 (28.8)	87 (46.8)	39 (36.1)	67 (39.2)	38 (36.2)
Group difference 95% CI (p-value)^3)^	0.89 to 3.88 (0.10)		0.62 to 2.43 (0.56)		0.99 to 2.94 (0.05)		0.74 to 2.16 (0.37)	

The spaces with “-“mean zero.

^1)^ Symptoms classified as warning signs to terminate exercise during pregnancy [3,4] and other symptoms reported by the participants (musculoskeletal symptoms, painless contractions, cramps and miscellaneous).

^2)^ N is one case less.

^3)^ Multilevel analysis (GLLAMM) taking into account the intra-level variation in pairs, municipalities and nurses and adjusting group differences for participants’ age, pre-pregnant body mass index (≤25, >25 kg/m^2^), smoking status before and/or during pregnancy (no/yes), primiparity (no/yes), education (academic, polytechnic, basic), working fulltime (no/yes) and weekly minutes of moderate-to-vigorous-intensity PA at baseline.

 The timing of the counseling sessions was as intended: The mean weeks’ gestation at the primary session was 9 (range 6 to 13), at the first booster session 17 (range 8-25), at the second booster session 23 (range 19 to 29), at the third booster session 33 (range 30 to 37) and at the finalbooster session 37 (range 34 to 40). The mean duration of the primary counseling session on PA was 21 min (range 5 to 55) and the duration of subsequent booster sessions 10 (range 0 to 30), 11 (range 2 to 32), 10 (range 0 to 56) and 6 min (range 2 to 20). Two participants missed the second booster session at 22-24 weeks’ gestation and three participants the last booster at 36-37 weeks’ gestation.

The average attendance at the monthly thematic meetings with group exercise was 33% ranging from 20% to 52% in the municipalities. On average, only 6% (municipality-specific range 0% to 15%) of the participants attended all thematic meetings and 33% (municipality-specific range 10% to 67%) at least 3 of the meetings during their pregnancy.

On a 5-point scale the applicability of the primary counseling session to the routine maternity care was scored 3.6 (SD 1.2) by the nurses. The corresponding score for booster visits was 4.6 (SD 0.5).

### Effectiveness

At the first follow-up at 26-28 weeks’ gestation, the decrease in the weekly total number of LTPA days from baseline was smaller in INT than in UC (0.1 vs. -1.2, p = 0.040) (Table 4). The finding was similar but slightly stronger in the weekly number of days with moderate-to- vigorous-intensity LTPA (−0.2 vs. -1.3, p = 0.016). Also, meeting the PA recommendation for health decreased less in INT (−11%-points) than in UC (−28%-points) although the difference in change between the groups did not quite reach statistical significance (p = 0.06). Other PA outcomes did not show statistical group differences from baseline to first follow-up. Neither were there any group differences from baseline to last follow-up at 36-37 weeks’ gestation. From 26-28 weeks’ gestation to 36-37 weeks’ gestation the number of weekly days with light-intensity LTPA decreased significantly less in INT than in UC (0.1 vs. 0.6 days, p = 0.05, not shown in Table 4).

**Table 4:** Unadjusted arithmetic means (SD) of weekly total leisure-time physical activity (LTPA), moderate-to-vigorous-intensity LTPA and light-intensity LTPA and percentage of participants meeting physical activity (PA) recommendations for health at baseline and two follow-ups in the intervention (INT) and usual care group (UC)

**N**	**Baseline**		**First follow-up**		**Last follow-up**		**Group difference in change from baseline to 26–28 weeks’ gestation**		**Group difference in change from baseline to 36–37 weeks’ gestation**		
	**Prior to pregnancy**		**26–28 weeks’ gestation**		**36–37 weeks’ gestation**						
	**INT**	**UC**	**INT**	**UC**	**INT**	**UC**	**Coeff./OR**	**95% CI**	**Coeff./OR**	**95% CI**	
	**215-218**	**167-174**	**212-215**	**178-179**	**190-192**	**156-158**	**p-value**		**p-value**		
**Total LTPA**										
Days/week	6.9 (3.5)	7.8^3)^ (3.8)	7.0 (3.6)	6.7 (3.6)	6.3 (3.5)	6.5 (3.5)	0.70^1)^	0.03 to 1.36	0.10^1)^	−0.70 to 0.91
							0.040		0.80	
Minutes/week	331 (298)	412 (315)	284 (210)	309 (292)	254 (227)	310 (289)	13.24	−33.20 to 56.67	−15.75	−75.15 to 43.65
							0.58		0.60	
**Moderate-to-vigorous LTPA**										
Days/week	3.6 (2.5)	4.3 (2.7)	3.4 (2.3)	3.1 (2.6)	2.6 (2.3)	2.4 (2.5)	0.56	0.10 to 1.01	0.36	−0.14 to 0.86
							0.016		0.16	
Minutes/week	169 (177)	212 (182)	133 (111)	132 (144)	96 (105)	101 (147)	18.21	−4.13 to 40.55	0.62	−25.59 to 26.83
							0.11		0.96	
**Light LTPA**										
Days/week	3.4 (2.5)	3.5 (2.3)	3.5 (2.2)	3.6 (2.3)	3.6 (2.5)	4.1 (2.3)	0.06	−0.37 to 0.48	−0.36	−0.93 to 0.20
							0.80		0.21	
Minutes/week	160 (219)	205 (252)	149 (171)	177 (213)	158 (189)	207 (201)	−8.47	−44.69 to 27.75	8.91	−45.27 to 63.10
							0.65		0.75	
**Meeting PA recommendations for health N (%)**^4)^	47	58	36	30	24	23	1.73^2)^	0.98 to 3.05	1.12^2)^	0.64 to 1.95
							0.06		0.70	

Group differences indicated as coefficients (Coeff.)^1)^ or odds ratios (OR)^2)^, 95% confidence intervals (95% CI) and statistical significance levels (p-value).

^1)^ Multilevel analysis (xtmixed) taking into account the intra-level variation in clusters (pairs), municipalities and nurses and adjusting group differences for participant’s baseline value of corresponding LTPA variable, age, BMI (≤25, >25 kg/m2), smoking status during pregnancy (no/yes), primiparity (no/yes), education (academic, polytechnic, basic) and working fulltime (no/yes).

^2)^ Multilevel analysis (GLLAMM) taking into account the intra-level variation in pairs, municipalities and nurses and adjusting group differences for participant’s baseline value of meeting the PA recommendation (no/yes), age, BMI (≤25, >25 kg/m2), smoking status before and/or during pregnancy (no/yes), primiparity (no/yes), education (academic, polytechnic, basic) and working fulltime (no/yes).

^3)^ Weekly number of days exceeds 7 because days of LTPA with various intensities have been summed up.

^4)^ The minimum of 150 min of moderate-to-vigorous-intensity LTPA spread throughout at least 3 days a week.

## Discussion

Intensified PA counseling supported with an option for monthly thematic meetings with group exercise proved feasible among pregnant women at risk for gestational diabetes and was able to reduce the decrease in their weekly frequency of total and moderate-to-vigorous- intensity LTPA until the end of the second trimester better than conventional counseling.

To the best of our knowledge this is the first study to report on the *safety* of PA counseling integrated into routine maternity care practices and involving pregnant women at risk for gestational diabetes. The findings indicated no more miscarriages or adverse events listed as warning signs to terminate exercise during pregnancy [3,4] in INT than in UC. Neither was there any difference in neonatal safety issues, which have been reported in more detail elsewhere [37]. The results are in line with studies reporting no harmful effects of moderate-intensity PA on pregnancy outcomes [13,14,47]. However, outside the list of warning signs, INT experienced throughout the pregnancy more musculoskeletal symptoms and painless contractions during or immediately after PA than UC. It is unlikely that the more frequent occurrences of these symptoms were exclusively due to the intervention because there was no between-group difference in change in the duration of total or moderate-intensity LTPA at either of the follow-ups and the only difference between the groups in the frequency of total and moderate-intensity LTPA was discovered at 26-28 weeks’ gestation. It is possible that some of the symptoms remained undetected in UC because the response rate to the questions related to the adverse events was lower in UC (on average 62.8%) than in INT (on average 86.0%). Nevertheless, the more profound reasons for the higher occurrence of musculoskeletal symptoms and painless contractions in INT as well as for the higher occurrence of dyspnea and painful contractions in UC remain unclear.

The high *realization of counseling* in terms of timing, duration and compliance shows that the intervention was carried out as intended, which improves the likelihood that the results are indeed due to the intervention. Assessing realization is essential especially in interventions delivered by a third party, implemented in a real world setting less controllable than laboratory surroundings and requiring a significant contribution from the deliverers to carry out the actions. To date no other studies have been reported on the realization of PA counseling in this specific target group.

*Attendance at monthly thematic meetings* on PA was disappointingly low since only a third of the participants attended at least three of the five meetings. A higher participation rate was expected because the meetings were arranged only once a month and considerable effort was made with SMS reminders and telephone feedback to encourage the women to attend the meetings. However, the percentage is similar to our pilot study, where 28% of the first time pregnant women participated in at least half of the group-exercise sessions arranged once a week during pregnancy [39]. Undoubtedly one reason for the low participation in the present study was the fact that many of the participants already had children making it difficult to find time for extra activities. In the study by Hui et al. [48] two thirds of the women attended at least three exercise sessions during pregnancy but more detailed information was not provided to enable comparison with our participation rate. To conclude, the attendance at group-exercise sessions offered in addition to maternal care was quite low. This could be improved after gaining more information about the reasons for non-participation and tailoring the exercise sessions accordingly.

*The nurses’ views on the applicability* of the counseling protocol were generally positive. The findings are similar to those of our pilot study [39] indicating that this kind of counseling on PA may be transferable to routine maternity care visits. The nurses’ views on applicability may also reflect their satisfaction at having a systematic tool for PA promotion. In a recent study by Stotland et al. [49] concerning counseling approaches in preventing excessive weight gain in prenatal care, health care providers felt unsure about the effectiveness of their counseling efforts and reported lack of training and tools for implementing counseling. Providers have also indicated a need for more information about the benefits and risks of PA during pregnancy [50]. According to our pilot study similar issues seem to apply to Finnish maternity care, where the counseling practices at baseline were surveyed (unpublished data). However, with the struggle of continuously diminishing resources, there may also be a need in maternity care for simpler and lighter approaches than the one examined in this study such as PA prescription, step-log monitoring, web-based programs or peer-support systems.

Regarding *LTPA during pregnancy* the findings concur with those of earlier studies indicating that women’s PA decreases during the course of pregnancy and tends to shift from moderate to lighter activities as the pregnancy proceeds [5,8]. The present study indicates that intensified counseling with an option for a monthly thematic meeting on PA can reduce the decrease in the weekly frequency of total and moderate-intensity LTPA compared to the conventional counseling until the end of the second trimester at 26-28 weeks’ gestation.

The finding is similar to that of our pilot study [39] with the exception that in the pilot study, where the follow-ups were at 16-18 and 37 weeks’ gestation, the statistical between-group difference was only discovered at 37 weeks’ gestation. However, the pilot study involved only first-time pregnant women not screened for specific health risks, whereas the present study also included multiparous women and exclusively women at risk for gestational diabetes. Therefore the women in the present study were slightly older, heavier and less physically active, presumably limiting their LTPA especially during the last trimester more than in the pilot study.

Nevertheless, in this study, the between-group difference in change at 26-28 weeks’ gestation was less than one day per week, 0.70 days in total LTPA and 0.56 days in moderate-to-vigorous-intensity LTPA, which seems quite modest from the prevention of gestational diabetes point of view especially since it was not sustained until the last trimester. Moreover, no effects were discovered in the weekly duration of total or intensity-specific weekly LTPA, which may be more crucial from the health perspective than the weekly frequency of LTPA. In this respect it may be that the changes in PA due to intervention had only limited potential to affect the incidence of gestational diabetes and the birthweight of the newborns, which were the two primary health outcomes of the whole lifestyle intervention [37]. However, the more precise timing as well as the amount and intensity of PA sufficient to prevent gestational diabetes need still to be determined in future studies.

### Strengths and limitations

The most important strength of the study was that the counseling was integrated into real maternity visits and implemented by the providers themselves. Together with feasibility evaluation this increases the pragmatic value of the findings and improves their transferability to practical maternity work. Counseling was also based on a behaviorally grounded model and the nurses were carefully trained and supported for implementation. In the analysis stage, multilevel models were used to reduce the bias related to intra-level variation within clusters, municipalities, nurses and individual participants.

The study nevertheless has some weaknesses to be taken into consideration when interpreting the results. Firstly, the representativeness of the study sample may have been impaired since there were fewer women whose BMI exceeded 25 and who were smokers in the final sample (N = 399) than among all eligible women (N = 726). The representativeness may also have been slightly although not notably hampered through the 29 dropouts, who had statistically lower education than the women in the final sample.

Secondly, the comparability of INT and UC may have suffered from the non-blinded allocation procedure, meaning that the participants were informed about the study group of their maternity clinic in the consent form. As shown in Table 4, the participants’ LTPA level at baseline was generally higher in UC than INT. This may have resulted from the higher refusal rate of women with low LTPA level in UC since the study offered them “no extra benefit”. In INT the situation may have been the opposite.

Thirdly, the power of the study had been calculated for the incidence of gestational diabetes, not for change in PA behavior. According to the calculations performed after the study the sample size was sufficient to discover the between-group difference of 40 min in the weekly duration of moderate-to-vigorous-intensity LTPA (intra-cluster correlation of 0.01, standard deviation of 125, significance level of 0.05 and power of 80%). Then, ideally, the number of participants in each municipality should have been 14. As the number of participants per municipality within the analyzed sample varied from 9 to 59 and the number of municipalities was quite minimal for multilevel models, this may have caused uneven weighing of data in the analysis.

Fourthly, bias may have resulted from using self-report as an outcome measure in PA assessment. It is possible that women in INT, being aware of the expectations related to their PA behavior, were more likely than women in UC to over-report their LTPA at the follow-ups. On the other hand, keeping a record of the realization of the action plans may have helped the participants in INT to recall their LTPA more accurately than their peers in UC. The use of RPE may also have improved the ability of the women in INT to classify the intensity of their LTPA in the follow-up questionnaires, which may have reduced the possible over-reporting of moderate-to-vigorous-intensity LTPA. In addition, recall errors may have occurred in assessing the LTPA prior to pregnancy since it was elicited at 8-12 weeks’ gestation. However, the possible recall errors at baseline were expected to be the same in INT and UC and should not have affected to group comparisons.

It can also be argued that the clinical significance of the findings concerning the weekly frequencies of total and intensity-specific LTPA should have been verified by complementing it with accurate information on between-group differences in the weekly duration of LTPA. However, the sensitivity of the self-report for detecting the differences was impaired in the sample of this size due to the wide variations in the weekly minutes of total and intensity-specific LTPA. In other words, the large individual differences in duration outcomes may have obscured the possible intervention effects. The assumption is, indeed, supported by the study by Aittasalo et al. [45], where larger random errors in duration than frequency estimates was discovered with regard to this particular questionnaire. In future studies, using more objective measures such as pedometers or accelerometers could diminish this deficit. Pedometer seems feasible among pregnant women [5] although the compliance may be lower in obese women [51]. In pregnant women the pedometer has also been shown to correlate with the exercise diary [52]. Studies on the feasibility and validity of using an accelerometer during pregnancy have not yet been published although the accelerometer has been used in pregnant women for validating PA self-reports [53] and assessing PA [54].

## Conclusions

The general PA recommendations for health are also valid during uncomplicated pregnancy and the importance of PA is especially emphasized among women at risk for gestational diabetes. However, women tend to decrease their PA and shift from moderate-intensity activities to light activities during pregnancy. In this study, intensified PA counseling supported by monthly group meetings on PA reduced the decrease in the frequency of total and moderate-to-vigorous-intensity LTPA until the end of the second trimester among pregnant women at risk for gestational diabetes. Counseling was safe in terms of warning signs to terminate exercise, was realized as intended except for the monthly group meetings and was viewed applicable to routine practices by the nurses. Further research is needed to confirm the findings with more objective PA measures and to compare this kind of counseling with approaches requiring lighter input from maternity staff, such as PA prescriptions, step-log monitoring, web-based programs or peer-support systems.

## Competing interest

The authors declare that they have no competing interests.

## Authors’ contributions

RL conceived and coordinated the NELLI study intended to prevent gestational diabetes, and participated in drafting the manuscript. MA, TIK and KO participated in the design of the NELLI study. MA designed the protocol, content and material for physical activity counseling and thematic meetings and is responsible for this manuscript. JR performed the statistical analysis and participated in drafting the manuscript. TIK helped in drafting the manuscript. KO planned the group exercise, analyzed adherence to the thematic meetings and participated in drafting the manuscript. PK helped in collecting, entering and analyzing the data and participated in drafting the manuscript. All authors have read and approved the final manuscript.

## References

[B1] Physical Activity Guidelines Advisory Committee Physical Activity Guidelines Advisory Committee Report, 2008 2008 U.S. Department of Health and Human Services, Washington DC http://www.health.gov/paguidelines/Report/pdf/CommitteeReport.pdf

[B2] Helakorpi S Laitalainen E Uutela A Health Behaviour and Health among the Finnish Adult Population, Spring 2009 National Institute for Health and Welfare (THL), Report 7/2010, 211 pages, Helsinki ISBN 978-952-245-231-3 (print), ISBN 978-952-245-232-0 (pdf). http://www.thl.fi/thl-client/pdfs/ce5ee5c1-6df4-44c2-bcd7-c3b735019570

[B3] ACOG (American College of Gynecologists) Committee, Opinion no 267 Exercise during pregnancy and the postpartum period Obstet Gynecol 2002 99 171 173 10.1016/S0029-7844(01)01749-5 11777528

[B4] Davies GAL Wolfe LA Mottola MF MacKinnon C Exercise in pregnancy and the postpartum period J Obstet Gynaecol Can 2003 25 516 522 29447726 10.1016/j.jogc.2017.11.001

[B5] Downs DS LeMasurier GC DiNallo JM Baby Steps: pedometer-determined and self-reported leisure-time exercise behaviors of pregnant women J Phys Act Health 2009 6 63 72 19211959 10.1123/jpah.6.1.63

[B6] Owe KM Nystad W Bø K Correlates of regular exercise during pregnancy: the Norwegian mother and child cohort study Scand J Med Sci Sports 2009 19 637 645 10.1111/j.1600-0838.2008.00840.x 18627550

[B7] Evenson KR Wen F National trends in self-reported physical activity and sedentary behaviors among pregnant women: NHANES 1999–2006 Prev Med 2010 50 123 128 10.1016/j.ypmed.2009.12.015 20053370

[B8] Pereira MA Rifas-Shiman SL Kleinman KP Rich-Edwards JW Peterson KE Gillman MW Predictors of change in physical activity during and after pregnancy Project Viva Am J Prev Med 2007 32 312 319 10.1016/j.amepre.2006.12.017 PMC1894953 17383562

[B9] Cramp AG Bray SR A prospective examination of exercise and barrier self-efficacy to engage in leisure-time physical activity during pregnancy Ann Behav Med 2009 37 325 334 10.1007/s12160-009-9102-y 19499279

[B10] Downs DS Hausenblas HA Women’s exercise beliefs and behaviors during their pregnancy and postpartum J Midwifery Womens Health 2004 49 138 144 15010667 10.1016/j.jmwh.2003.11.009

[B11] Sampselle CM Seng J Yeo S Killion C Oakley D Physical activity and postpartum wellbeing JOGNN 1999 28 41 49 10.1111/j.1552-6909.1999.tb01963.x 9924863

[B12] Domingues MR Matijasevitch A Barros AJ Physical activity and preterm birth: a literature review Sports Med 2009 39 961 975 10.2165/11317900-000000000-00000 19827862

[B13] Gavard JA Artal R Effect of exercise on pregnancy outcome Clin Obstet Gynecol 2008 51 467 480 10.1097/GRF.0b013e31816feb1d 18463475

[B14] Hegaard HK Pedersen BK Nielsen B Damm P Leisure- time physical activity during pregnancy and impact on gestational diabetes mellitus, pre-eclampsia, preterm delivery and birth weight: a review Acta Obstet Gynecol Scand 2007 86 1290 1296 10.1080/00016340701647341 17851805

[B15] Melzer K Schutz Y Boulvain M Kayser B Physical activity and pregnancy: cardiovascular adaptations, recommendations and pregnancy outcomes Sports Med 2010 40 493 507 10.2165/11532290-000000000-00000 20524714

[B16] Pivarnik JM Chambliss HO Clapp JF Dugan SA Hatch MC Lovelady CA Mottola MF Williams MA Impact of physical activity during pregnancy and postpartum on chronic disease risk Med Sci Sports Exerc 2006 38 989 1006 10.1249/01.mss.0000218147.51025.8a 16672855

[B17] Weissgerber TL Wolfe LA Davies GAL Mottola MF Exercise in the prevention and treatment of maternal-fetal disease: a review of the literature Appl Physiol Nutr Metab 2006 31 661 674 10.1139/h06-060 17213880

[B18] Brown W The benefits of physical activity during pregnancy J Sci Med Sport 2002 5 37 45 12054385 10.1016/s1440-2440(02)80296-1

[B19] Ratner RE Christophi CA Metzger BE Dabelea D Bennett PH Pi-Sunyer X Fowler S Kahn SE The Diabetes Prevention Program Research Group Prevention of diabetes in women with a history of gestational diabetes: effects of metformin and lifestyle interventions J Clin Endocrinol Metab 2008 93 4774 4779 10.1210/jc.2008-0772 18826999 PMC2626441

[B20] NICE (National Institute for Health and Clinical Excellence) Diabetes in pregnancy. management of diabetes and its complications from pre-conception to the postnatal period 2008 http://www.nice.org.uk/nicemedia/live/11946/41321/41321.PDF

[B21] Tobias DK Zhang C van Dam RM Bowers K Hu FB Physical activity before and during pregnancy and risk of gestational diabetes mellitus: a meta-analysis Diabetes Care 2011 34 223 229 10.2337/dc10-1368 20876206 PMC3005457

[B22] Paisley TS Joy EA Price RJ Exercise during pregnancy: a practical approach Curr Sports Med Rep 2003 2 325 330 14583162 10.1249/00149619-200312000-00008

[B23] Phelan S Pregnancy: a “teachable moment” for weight control and obesity prevention Am J Obstet Gynecol 2010 202 e1 e8 10.1016/j.ajog.2009.06.008 PMC2815033 19683692

[B24] Duncombe D Wertheim EH Skouteris H Paxton SJ Kelly L Factors related to exercise over the course of pregnancy including women’s beliefs about the safety of exercise during pregnancy Midwifery 2009 25 430 438 10.1016/j.midw.2007.03.002 18063253

[B25] Haakstad LA Voldner N Henriksen T Bø K Why do pregnant women stop exercising in the third semester? Acta Obstet Gynecol Scand 2009 88 1267 1275 10.3109/00016340903284901 19824869

[B26] Evenson KR Bradley CB Beliefs about exercise and physical activity among pregnant women Patient Educ Couns 2010 79 124 129 10.1016/j.pec.2009.07.028 19699603 PMC2848492

[B27] Mudd LM Nechuta S Pivarnik JM Paneth N Factors associated with women’s perceptions of physical activity safety during pregnancy Prev Med 2009 49 194 199 10.1016/j.ypmed.2009.06.004 19540874

[B28] National Institute for Health and Welfare Births and newborns 2008.Statistical report 22 2009 http://www.stakes.fi/tilastot/tilastotiedotteet/2009/tr22_09.pdf

[B29] Stakes (National Research and Development Centre for Welfare and Health) Parturients, Deliveries and Births 2006 2007 http://www.stakes.fi/FI/Tilastot/Ai-heittain/Lisaantyminen/synnyttajat/index.htm

[B30] Callaway LK Colditz PB Byrne NM Lingwood BE Rowlands IJ Foxcroft K McIntyre HD Bambino Group Prevention of gestational diabetes: feasibility issues for an exercise intervention in obese pregnant women Diabetes Care 2010 33 1457 1459 10.2337/dc09-2336 20357374 PMC2890340

[B31] Gray-Donald K Robinson E Collier A David K Renaud L Rodrigues S Intervening to reduce weight gain in pregnancy and gestational diabetes mellitus in Cree communities: an evaluation CMAJ 2000 163 1247 1251 11107459 PMC80308

[B32] Mottola MF Kander S Giroux I Hammond J-A Lebrun C McManus R Sopper MM Glucose and insulin responses in women at risk for GDM before and after a Nutrition, Exercise & Lifestyle Intervention Program (NELIP) Med Sci Sports Exerc 2005 37 S309 S310

[B33] Oostdam N van Poppel MNM Eekhoff EMW Wouters MGAJ van Mechelen W Design of FitFor2 study: the effects of an exercise program on insulin sensitivity and plasma glucose levels in pregnant women at high risk for gestational diabetes BMC Pregnancy and Childbirth 2009 9 1 10.1186/1471-2393-9-1 19123930 PMC2649039

[B34] Oostdam N van Poppel MN Wouters MG van Mechelen W Interventions for preventing gestational diabetes mellitus: a systematic review and meta-analysis J Womens Health (Larchmt) 2011 20 1551 1563 10.1089/jwh.2010.2703 21838525

[B35] Chasan-Taber L Marcus B Stanek E Ciccolo JT Marquez DX Solomon CG Markenson G A randomized controlled trial of prenatal physical activity to prevent gestational diabetes: design and methods J Women’s Health 2009 18 851 859 10.1089/jwh.2008.1006 PMC2851124 19514827

[B36] Luoto RM Kinnunen TI Aittasalo M Ojala K Mansikkamäki K Toropainen E Kolu P Vasankari T Prevention of gestational diabetes: design of a cluster-randomized controlled trial and one-year follow-up BMC Pregnancy Childbirth 2010 10 39 10.1186/1471-2393-10-39 20682023 PMC2923097

[B37] Luoto RM Kinnunen TI Aittasalo M Kolu P Raitanen J Ojala K Mansikkamäki K Lamberg S Vasankari T Komulainen T Tulokas S Primary prevention of gestational diabetes mellitus and large-for-gestational-age newborns by lifestyle counseling: a cluster randomized controlled trial PLoS Med 2011 8 e1001036 10.1371/journal.pmed.1001036 21610860 PMC3096610

[B38] Laitakari J Asikainen TM How to promote physical activity through individual counseling — a proposal for a practical model of counseling on health-related physical activity Patient Educ Couns 1998 33 S13 S23 10889742 10.1016/s0738-3991(98)00005-6

[B39] Aittasalo M Pasanen M Fogelholm M Kinnunen TI Ojala K Luoto R Physical activity counseling in maternity and child health care — a controlled trial BMC Women's Health 2008 8 14 10.1186/1472-6874-8-14 18702803 PMC2527301

[B40] Borg GA Psychophysical bases of perceived exertion Med Sci Sports Exerc 1982 14 377 381 7154893

[B41] Artal R O’Toole M Guidelines of the american college of obstetricians and gynecologists for exercise during pregnancy and the postpartum period Br J Sports Med 2003 37 6 12 10.1136/bjsm.37.1.6 12547738 PMC1724598

[B42] Pate RR Pratt M Blair SN Haskell WL Macera CA Bouchard C Buchner D Ettinger W Heath GW King AC Kriska A Leon AS Marcus BH Morris J Paffenbarger RS jr Patrick K Pollock ML Rippe JM Sallis J Wilmore JH Physical activity and public health. A recommendation from the Centers for disease control and prevention and the American College of Sports Medicine JAMA 1995 273 402 407 10.1001/jama.1995.03520290054029 7823386

[B43] Haskell WL Lee I-M Pate RR Powell KE Blair SN Franklin BA Macera CA Heath GW Thompson PD Bauman A Physical activity and public health. updated recommendation for adults from the American College of Sports medicine and the American Heart Association Circulation 2007 116 1081 1093 17671237 10.1161/CIRCULATIONAHA.107.185649

[B44] Howley ET Type of activity: resistance, aerobic and leisure versus occupational physical activity Med Sci Sports Exerc 2001 33 S364 S369 10.1097/00005768-200106001-00005 11427761

[B45] Aittasalo M Pasanen M Fogelholm M Ojala K Validity and repeatability of a short pregnancy leisure time physical activity questionnaire J Phys Act Health 2010 7 109 118 20231762 10.1123/jpah.7.1.109

[B46] Tudor-Locke C Henderson KA Wilcox S Cooper RS Durstine JL Ainsworth BE In their own voices: definitions and interpretations of physical activity Women’s Health Issues 2003 13 194 199 10.1016/S1049-3867(03)00038-0 14583168

[B47] Melzer K Schutz Y Soehnchen N Othenin-Girard V de TB M Irion O Boulvain M Kayser B Effects of recommended levels of physical activity on pregnancy outcomes Am J Obstet Gynecol 2010 202 e1 e6 20022583 10.1016/j.ajog.2009.10.876

[B48] Hui AM Ludwig SM Gardiner P Sevenhuysen G Murray R Morris M Shen GX Community-based exercise and dietary intervention during pregnancy: a pilot study Can J Diabetes 2006 30 169 175

[B49] Stotland NE Gilbert P Bogetz A Harper CC Abrams B Gerbert B Preventing excessive weight gain in pregnancy: how do prenatal care providers approach counseling? J Womens Health 2010 19 807 814 10.1089/jwh.2009.1462 PMC2867592 20078239

[B50] Evenson KR Pompeii LA Obstetrician practice patterns and recommendations for physical activity during pregnancy J Womens Health (Larchmt) 2010 19 1733 1740 10.1089/jwh.2009.1833 20718678

[B51] Renault K Nørgaard K Andreasen KR Secher NJ Nilas L Physical activity during pregnancy in obese and normal-weight women as assessed by pedometer Acta Obstet Gynecol Scand 2010 89 956 961 10.3109/00016341003792459 20583938

[B52] Lindseth G Vari P Measuring physical activity during pregnancy West J Nurs Res 2005 27 722 734 10.1177/0193945905276523 16157944

[B53] Chasan-Taber L Schmidt MD Roberts DE Hosmer D Markenson G Freedson PS Development and validation of a pregnancy physical activity questionnaire Med Sci Sports Exerc 2004 36 1750 1760 10.1249/01.MSS.0000142303.49306.0D 15595297

[B54] Rousham EK Clarke PE Gross H Significant changes in physical activity among pregnant women in the UK as assessed by accelerometry and self-reported activity Eur J Clin Nutr 2006 60 393 400 10.1038/sj.ejcn.1602329 16306930

